# Interventional treatment combined with immunotargeted therapy in unresectable combined hepatocellular-cholangiocarcinoma: a real-world retrospective cohort study

**DOI:** 10.3389/fimmu.2025.1591127

**Published:** 2025-07-01

**Authors:** Yan-Song Lin, Li-Yan Wu, Li-Hui Lin, Xia Yang, Fang-Yi Liu, Yan-Qin Wu, Zhen Ding, Yu-Jing Liang, Jing-Ping Yun

**Affiliations:** ^1^ State Key Laboratory of Oncology in South China, Guangdong Provincial Clinical Research Center for Cancer, Sun Yat-sen University Cancer Center, Guangzhou, China; ^2^ Department of Pathology, Sun Yat-sen University Cancer Center, Guangzhou, China; ^3^ Department of Gastroenterology, The First Affiliated Hospital, Sun Yat-sen University, Guangzhou, China; ^4^ Department of Interventional Oncology, The First Affiliated Hospital, Sun Yat-sen University, Guangzhou, China; ^5^ Department of Radiology, Sun Yat-sen University Cancer Center, Guangzhou, China

**Keywords:** interventional treatment, PD-(L)1 inhibitor, targeted therapy, unresectable combined hepatocellular-cholangiocarcinoma, outcome

## Abstract

**Background:**

Evidence-based treatment for unresectable combined hepatocellular-cholangiocarcinoma (cHCC-CCA) has not been established. This study aimed to assess the effectiveness and safety of interventional treatment combined with immunotargeted therapy (IIT) in unresectable cHCC-CCA patients.

**Methods:**

Patients with a histological diagnosis of unresectable cHCC-CCA who received IIT therapy from January 2019 to March 2024 were retrospectively enrolled. The study evaluated overall survival (OS), progression-free survival (PFS), tumor responses and safety.

**Results:**

A total of 242 cHCC-CCA patients were screened and 51 patients were enrolled for analysis. The median follow-up duration was 15.8 months (95% CI: 12.0-19.7 months). The median OS was 17.8 months (95% CI: 12.4-23.2 months) and the median PFS was 8.9 months (95% CI: 5.8-12.0 months). For overall response, the objective response rate was 41.2% and 56.9% based on RECIST 1.1 and mRECIST, respectively. Patients with primary cHCC-CCA showed significantly prolonged OS (median OS: 21.4 months vs. 11.4 months, *p* = 0.011) and PFS (median PFS: 9.5 months vs. 4.1 months, *p* = 0.036) compared to those with recurrent cHCC-CCA. Patients with dominant HCC did not show significant differences for OS (*p* = 0.835) and PFS (*p* = 0.553) compared to those with dominant iCCA. Six patients (11.8%) experienced grade ≥3 adverse events, including leukopenia (n=1, 2.0%), neutropenia (n=1, 2.0%), thrombocytopenia (n=2, 3.9%), elevated alanine transaminase (ALT) (n=2, 3.9%), elevated aspartate aminotransferase (AST) (n=2, 3.9%), hypoalbuminemia (n=2, 3.9%), and hyperbilirubinemia (n=1, 2.0%). Immunotherapy was discontinued for two patients due to grade ≥3 elevations in ALT and AST.

**Conclusion:**

The triple combination of interventional treatment, PD-(L)1 inhibitor, and targeted therapy is an effective and safe approach for unresectable cHCC-CCA patients.

## Highlights

- Combined hepatocellular-cholangiocarcinoma (cHCC-CCA) is a rare cancer without specific evidence-based treatments.
- This study reported the efficacy and safety of a triple combination therapy for managing unresectable cHCC-CCA.
- This triple combination therapy had favorable survival benefits and controllable adverse events for unresectable cHCC-CCA.

## Introduction

Combined hepatocellular-cholangiocarcinoma (cHCC-CCA) is a rare form of cancer, comprising 0.4%-14.2% of primary liver cancer (PLC) (1, 2). The cHCC-CCA exhibits both hepatocytic and cholangiocytic differentiation, contributing to its heterogeneous nature. Due to its rarity and complexity, cHCC-CCA presented diagnostic and therapeutic challenges. The definition of cHCC-CCA was updated in the World Health Organization (WHO) Classification of Tumors of the Digestive System (3). Surgical resection and liver transplantation offer curative potential for localized disease, while locoregional therapies or systemic treatments are recommended for patients with unresectable disease.

Currently, there is no established evidence-based treatment specifically for cHCC-CCA patients, leading to extrapolation from hepatocellular carcinoma (HCC) or intrahepatic cholangiocarcinoma (iCCA) regimens. Some retrospective studies have reported the clinical efficacy of tyrosine kinase inhibitors (TKIs) and platinum‐based chemotherapy for cHCC-CCA, which were commonly recommended for HCC and iCCA, respectively. A multicenter study involving 30 patients treated with gemcitabine plus oxaliplatin (GEMOX), GEMOX plus bevacizumab or gemcitabine plus cisplatin, indicated a median progression‐free survival (PFS) of 9.0 months and an overall survival (OS) of 16.2 months (4). Gignate et al. reported that TKI therapy and platinum-based chemotherapy demonstrated similar efficacy in patients with cHCC-CCA, with a median OS of 8.3 months in the TKI group compared to 11.9 months in the platinum-based chemotherapy group (5).

In recent years, immune checkpoint inhibitors (ICIs) have shown promising clinical outcomes in patients with unresectable HCC and iCCA. The combinations of atezolizumab and bevacizumab, sintilimab and bevacizumab, camrelizumab and apatinib have been recommended as first‐line therapies for unresectable HCC in China (6–9). A phase III study, TOPAZ-1, indicated that the combination of durvalumab with gemcitabine and cisplatin significantly improved median OS and PFS in patients with advanced biliary tract cancer compared to gemcitabine and cisplatin alone (10). Elia Gigante reported that patients with non‐resectable or metastatic cHCC-CCA who received first-line atezolizumab and bevacizumab achieved a median OS of 13.0 months and a median PFS of 3.0 months (11). Interventional treatments, transarterial chemoembolization (TACE) and hepatic arterial infusion chemotherapy (HAIC) with oxaliplatin, fluorouracil, and leucovorin (FOLFOX) have demonstrated favorable efficacy and safety in advanced HCC and iCCA, especially when combined with ICIs and TKIs (12–14). HAIC or TACE can activate systemic immune responses by enhancing the maturation and function of dendritic cells and T cells or mitigating immune function inhibition (15–17). Considering the underlying synergic effect of combining interventional treatment, immunotherapy, and molecular targeted therapy, we hypothesize that triple-modality therapy may overcome the limitations of current strategies.

Herein, we conducted this study to evaluate the clinical outcomes, tumor responses and safety of patients with unresectable cHCC-CCA receiving interventional treatment in conjunction with immunotargeted therapy.

## Materials and methods

### Study design and patients

In this retrospective study, patients diagnosed with unresectable cHCC-CCA who received interventional treatment plus ICI therapy and molecular targeted therapy were consecutively enrolled at Sun Yat-Sen University Cancer Center from January 2019 to March 2024. The study was approved by the Institutional Review Board of Sun Yat-sen University Cancer Center (Approval No.: B2024-454-01) and performed in accordance with Declaration of Helsinki of 1975, as revised in 1983. Written informed consent for treatment was obtained from all enrolled patients. The inclusion criteria for the study were as follows: 1) patients aged 18–80 years old; 2) histological diagnosis of cHCC‐CCA according to the 2018 WHO classification; 3) deemed unresectable by hepatobiliary specialists due to multifocal disease, vascular invasion, lymph node/distant metastases or technical inoperability; 4) received first-line therapy with TACE and/or HAIC combined with ICI therapy and molecular targeted therapy; 5) measurable and evaluable lesions; 6) Eastern Cooperative Oncology Group (ECOG) performance status 0-1; 7) Child-Pugh class A-B. Patients were excluded based on the following exclusion criteria: 1) concurrent malignancies; 2) received other anticancer therapies; 3) incomplete clinical or follow-up data.

### Treatment procedures

The interventional treatment comprised TACE, HAIC or a combination of both TACE and HAIC (TACE-HAIC). TACE and HAIC were conducted following previously reported protocols (18–20). For TACE, chemoembolization was performed using 30 mg/m^2^ of epirubicin, 200 mg/m^2^ of carboplatin, and 4 mg/m^2^ of mitomycin C, mixed with 2–5 mL lipiodol. An appropriate amount of pure lipiodol (not exceed 20 mL) were injected into the feeding artery of the tumors until the stasis of blood flow was observed. TACE was repeated at intervals of 3 to 4 weeks (18). For HAIC, on the first day of the treatment cycle, a femoral artery puncture was performed to place a catheter into the artery. The catheter was intubated to the predesigned position of hepatic artery. Following this procedure, the patient was transferred to the ward, where the catheter was connected to an infusion pump for drug administration according to the FOLFOX regimen: 85mg/m^2^ of oxaliplatin, 400 mg/m^2^ of leucovorin, and 400 mg/m^2^ of fluorouracil on the first day, followed by an additional 2400 mg/m^2^ of fluorouracil administered over 46 hours. HAIC cycle was performed every 3 weeks (19, 20). The TACE-HAIC procedure has been described in detail in a previous study (21). Initially, chemoembolization was performed using only 30mg/m^2^ of epirubicin mixed with 2–5 mL lipiodol, followed by pure lipiodol. Then, a catheter was placed and secured in the tumor’s feeding artery for the FOLFOX-based chemotherapy infusion, which involved: 85 mg/m^2^ of oxaliplatin infused over 2 hours; 400 mg/m^2^ of leucovorin infused over 2 hours; a bolus of 400 mg/m^2^ of 5-FU, and either a continuous infusion of 2400 mg/m² of fluorouracil over 46 hours or 1200 mg/m² of continuous fluorouracil over 23 hours. TACE-HAIC was repeated at intervals of 3 to 4 weeks. ICI therapy included one programmed cell death protein 1 (PD-1) antibody (tislelizumab, sintilimab, toripalimab, pembrolizumab) or one programmed death ligand-1 (PD-L1) antibody (camrelizumab, atezolizumab, durvalumab). Targeted therapy included one molecular targeted drug (lenvatinib, apatinib, sorafenib, bevacizumab). Standard doses and frequencies of anti-PD-(L)1 agents and molecular targeted agents were administrated (**Supplementary Table 1**). Anti-PD-(L)1 therapy was administrated at least three days before or after interventional therapy. The oral molecular targeted drug was administrated within two weeks before or after interventional therapy or anti-PD-(L)1 agent, while bevacizumab was administrated concurrently with the anti-PD-(L)1 agent. Treatments were discontinued due to disease progression, intolerable toxicity, or patient’s own choice. For patients undergoing hepatic resection after tumor downstaging, the continuation of anti-PD-(L)1 agents and targeted agents as adjuvant therapy was determined by a multidisciplinary team of hepatobiliary surgeons, radiologists and pathologists. The physicians would inform the patient about the advantages and disadvantages of adjuvant therapy, including potential therapeutic effects, adverse events, and associated costs. The final decision rested with the patient.

### Data collection and efficacy assessments

Clinical and radiological data were retrospectively collected from medical records. The demographic and clinical data included age, sex, hepatitis B surface antigen (HbsAg), ECOG performance status, white blood cell count (WBC), platelet count (PLT), albumin (ALB), alanine transaminase (ALT), aspartate aminotransferase (AST), total bilirubin (TBIL), C-reactive protein (CRP), liver function grade (Child-Pugh), α-fetoprotein (AFP), protein induced by vitamin K absence or antagonist II (PIVKA-II), carbohydrate antigen 19-9 (CA19-9), longest tumor diameter, tumor number, macroscopic vein invasion, lymph node metastasis, distant metastasis, and tumor-node-metastasis (TNM) stage. The dominant tumor type, either HCC or iCCA, was defined as the case in which one component constituted at least 20% more than the other (22). Tumor response was evaluated based on computed tomography (CT) and magnetic resonance imaging (MRI), following the Response Evaluation Criteria In Solid Tumors version 1.1 (RECIST 1.1) and modified RECIST (mRECIST) (23, 24), at a 4–6 weeks interval after initial treatment and a 8 week interval subsequently (25). Tumor responses were categorized as complete response (CR), partial response (PR), stable disease (SD), or progressive disease (PD). PD was defined as ≥20% increase in sum of target lesion diameters (or new lesions), according to mRECIST. The objective response rate (ORR) was calculated as the sum of CR and PR, while the disease control rate (DCR) encompassed CR, PR, and SD. The primary endpoint was PFS. The secondary outcomes included OS, ORR, and safety. PFS was defined as the duration from first-line treatment initiation to disease progression or the last follow-up date. OS was defined as the time interval from the commencement of first-line treatment to cancer-related death or the last follow-up. The follow-up deadline was July 31^st^, 2024, and the survival status of all patients were updated accordingly. Treatment-related adverse events (AEs) were assessed according to the National Cancer Institute Common Terminology Criteria for Adverse Events (CTCAE) version 5.0 (26).

### Statistical analysis

Normally distributed variables were reported as the means and standard deviations, while non-normally distributed variables were expressed as the medians and quartiles. Binary variables were expressed as number and proportion. Survival analysis was conducted using the Kaplan-Meier method, with differences in survival curves evaluated through the log-rank test. Variables demonstrating a univariate p-value of less than 0.05, or those deemed potentially impactful to patient prognosis, were included in a multivariate Cox proportional hazards regression analysis. All analyses were performed using SPSS 25.0 software (SPSS Inc., Chicago, IL) and GraphPad Prism version 8.0 (GraphPad, Inc.). A two-tailed p-value of <0.05 was considered statistically significant.

## Results

### Patients

**Figure 1:**
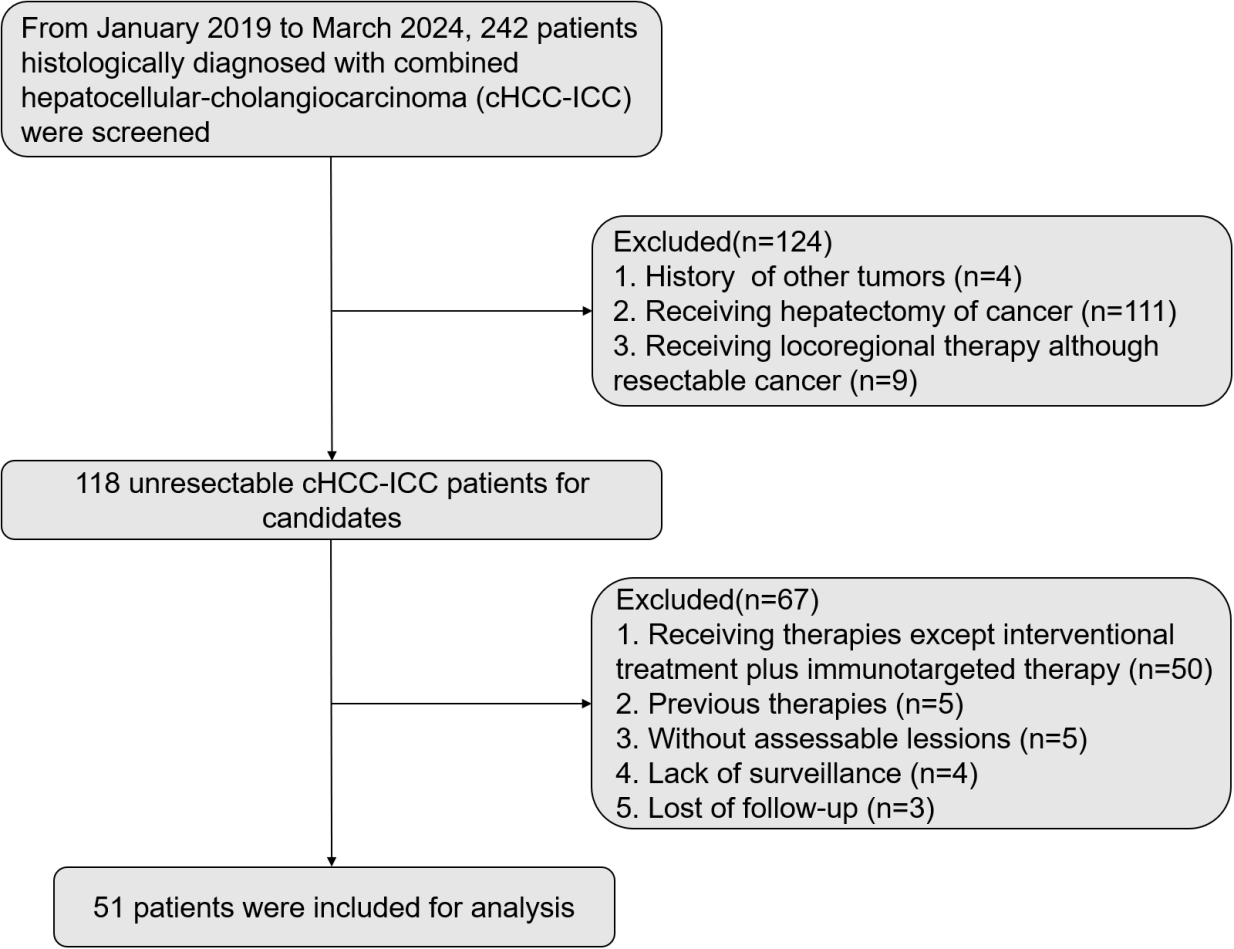
Flow diagram for patient inclusion.

To assess the efficacy and safety of this triple therapy, we screened a total of 242 patients histologically diagnosed with cHCC-CCA, from January 2019 to March 2024. Among these patients, 4 patients had a history of other tumors; 111 patients underwent hepatectomy for cancer; 9 patients received locoregional therapy despite having resectable cancer; 50 patients received therapies other than interventional treatment plus immunotargeted therapy; 5 patients participated in other treatments previously; 5 patients didn’t have assessable lesions; 4 patients lacked adequate medical surveillance; 3 patients lost to follow-up. Ultimately, 51 patients with unresectable cHCC-CCA who received first-line TACE and/or HAIC in combination with one immune checkpoint inhibitor and one targeted drug were enrolled. The details of patient inclusion process were delineated in **Figure 1**. The deadline of data collection was July 31^st^, 2024.

The baseline characteristics were summarized in **Table 1**. The representative pathological picture of cHCC-CCA was displayed in **Supplementary Figure 1**. The patient population were predominantly male (96.1%), with a mean age of 52.4 ± 10.4 years. Notably, 80.4% of the patients tested positive for HbsAg, and all patients were classified as ECOG 0–1 and Child-Pugh A. Elevated levels were observed in 37.3% of patients for AFP (≥400 ng/mL), 70.6% for PIVKA-II (>40 mAU/mL), and 45.1% for CA 19-9 (>35 U/mL), respectively. In terms of tumor composition, 29 patients (56.9%) had dominant HCC, while 19 patients (37.3%) exhibited dominant iCCA. The median size of the largest tumor nodule was 8.2 cm (interquartile range [IQR]: 3.7-10.9 cm), and most of the patients (82.4%) had multiple tumors. Additionally, 26 patients (51.0%) had lymph node metastasis and 13 patients (25.5%) had distant metastasis. Advanced TNM staging was prevalent, as 62.7% (32/51) of patients were stage IV, while an additional 29.4% (15/51) were stage III. Tumor downstaging followed by hepatic resection was performed in 11 patients (21.6%). The categories of the interventional treatment combined with immunotargeted therapy were summarized in **Supplementary Table 2**. The median number cycles for interventional therapy and immunotherapy was 3 (IQR: 2-5) and 5 (IQR: 3-9), respectively, while the median duration of targeted therapy was 5.8 months (IQR: 3.0-11.7 months).

**Table 1:** Baseline characteristics.

Variables	N=51
Age (years)	52.4 ± 10.4
Sex	
Male	49 (96.1)
Female	2 (3.9)
HbsAg, n (%)	
Positive	41 (80.4)
Negative	10 (19.6)
ECOG	
0	30 (58.8)
1	21 (41.2)
Child-Pugh	
5	38 (74.5)
6	13 (25.5)
ALB (g/L)	41.3 ± 5.2
ALT (U/L)	32.7 (23.9-47.6)
AST (U/L)	44.0 (33.3-60.7)
GGT (U/L)	155.0 (81.0-200.0)
TBIL (μmol/L)	13.3 (10.0-18.3)
CRP (mg/L)	9.7 (3.6-21.2)
AFP (ng/ml)	
<400	32 (62.7)
≥400	19 (37.3)
PIVKA-II (mAU/ml)	
≤40	15 (29.4)
>40	36 (70.6)
CA 19-9 (U/ml)	
≤35	28 (54.9)
>35	23 (45.1)
Liver cirrhosis	
Present	31 (60.8)
Absent	20 (39.2)
Composition of the tumor^#^	
HCC dominant	29 (56.9)
iCCA dominant	19 (37.3)
Largest nodule size (cm)	8.2 (3.7-10.9)
Tumor number	
Solitary	9 (17.6)
Multiple	42 (82.4)
Macroscopic vein invasion	
Present	24 (47.1)
Absent	27 (52.9)
Lymph node metastasis	
Present	26 (51.0)
Absent	25 (49.0)
Distant metastasis	
Present	13 (25.5)
Absent	38 (74.5)
TNM stage	
II	4 (7.8)
III	15 (29.4)
IV	32 (62.7)
Status of disease	
Primary cHCC-CCA	37 (72.5)
Recurrent cHCC-CCA	14 (27.5)
Conversion to resection	
Yes	11 (21.6)
No	40 (78.4)

Values are presented as means ± standard deviation, median (range) or n (%). ^#^Three patients were classified as neither HCC dominant nor iCCA dominant.

HBsAg, hepatitis B surface antigen; ECOG, Eastern Cooperative Oncology Group; ALB, albumin; ALT, alanine transaminase; AST, aspartate transaminase; GGT, gamma-glutamyl transpeptidase; TBIL, total bilirubin; CRP, C-reactive protein; AFP, alpha-fetoprotein; PIVKA-II, protein induced by vitamin K absence or antagonist-II; CA19-9, carbohydrate antigen 19-9; HCC, hepatocellular carcinoma; iCCA, intrahepatic cholangiocarcinoma; TNM, tumor–node–metastasis; cHCC-CCA, combined hepatocellular-cholangiocarcinoma.

### Treatment efficacy and patient survival

**Figure 2:**
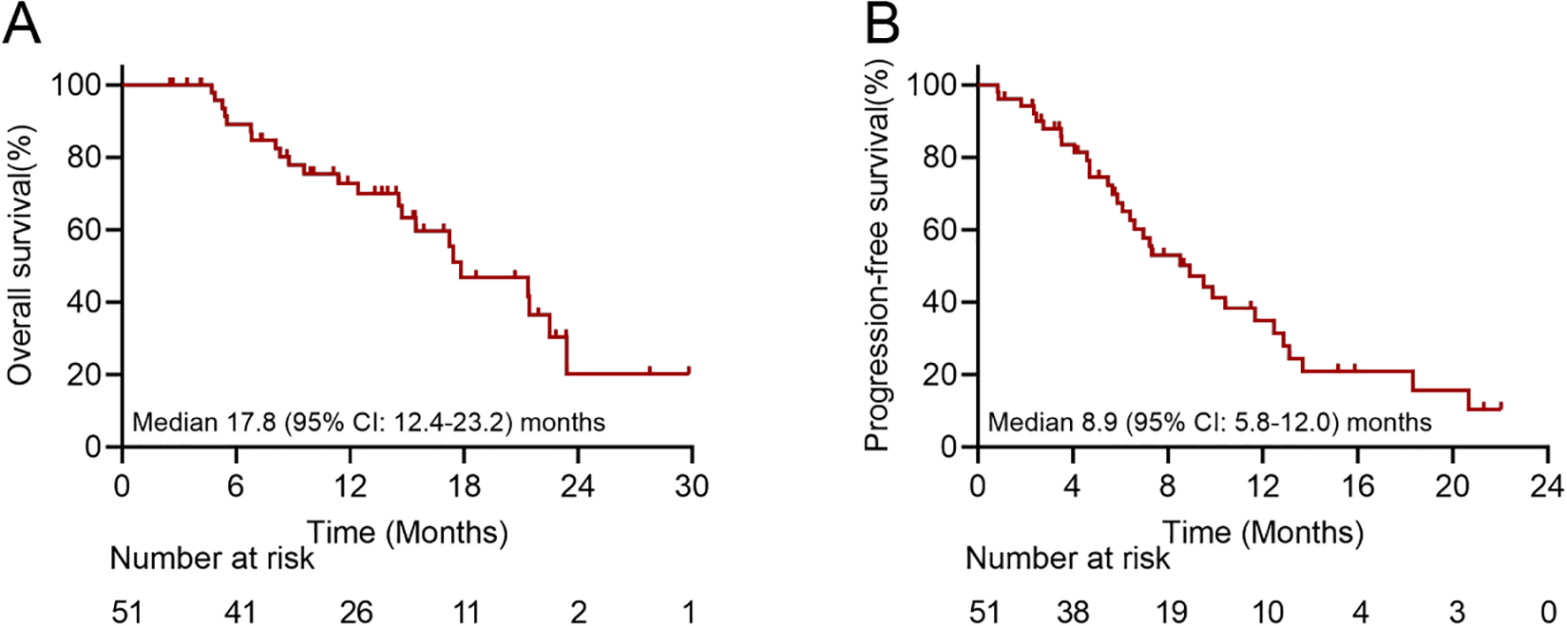
Overall survival and progression-free survival in patients treated with first-line interventional treatment combined with immunotargeted therapy. **(A)** Overall survival was evaluated by Kaplan‐Meier curve. The median overall survival was 17.8 months. **(B)** Progression-free survival was evaluated by Kaplan‐Meier curve. The median progression-free survival was 8.9 months. CI, confidence interval.

The median follow-up duration was 15.8 months (95% confidence interval [CI]: 12.0-19.7 months). Based on RECIST 1.1, the ORR were 45.1% for intrahepatic response and 41.2% for overall response, while the DCR were 92.2% and 78.4% for intrahepatic response and overall response, respectively. According to mRECIST, 3 patients (5.9%) achieved CR and 27 patients (52.9%) achieved PR in intrahepatic response, resulting in an ORR of 58.8% and a DCR of 92.2%. For overall response, 1 patient (2.0%) achieved CR and 28 patients (54.9%) achieved PR in overall response, leading to an ORR of 56.9% and a DCR of 78.4% (**Table 2**). The best response for intrahepatic lesions according to RECIST1.1 and mRECIST were illustrated in the waterfall plot in **Supplementary Figure 2**. During follow-up, 33 patients (64.7%) experienced radiological disease progression, and 23 patients (45.1%) died. The median intrahepatic PFS reached 18.2 months (95% CI: 12.5-23.8 months), while the overall cohort showed a median PFS of 8.9 months (95% CI: 5.8-12.0 months) and a median OS of 17.8 months (95% CI: 12.4-23.2 months) (**Figure 2**; **Supplementary Figure 3**). The 6-month, 1-year and 2-year OS rates were 89.1%, 70.1% and 20.3%, respectively. The 6-month and 1-year PFS were 67.5% and 34.5%, respectively (**Table 2**).

**Table 2:** Tumor responses and survival.

Responses	RECIST 1.1	mRECIST
Intrahepatic response		
CR, n (%)	0 (0.0)	3 (5.9)
PR, n (%)	23 (45.1)	27 (52.9)
SD, n (%)	24 (47.1)	17 (33.4)
PD, n (%)	4 (7.8)	4 (7.8)
ORR (CR+PR), n (%)	23 (45.1)	30 (58.8)
DCR (CR+PR+SD), n (%)	47 (92.2)	47 (92.2)
Overall response		
CR, n (%)	0 (0.0)	1 (2.0)
PR, n (%)	21 (41.2)	28 (54.9)
SD, n (%)	19 (37.3)	11 (21.6)
PD, n (%)	11 (21.6)	11 (21.6)
ORR (CR+PR), n (%)	21 (41.2)	29 (56.9)
DCR (CR+PR+SD), n (%)	40 (78.4)	40 (78.4)
Median follow-up, (month)	15.8 (95% CI: 12.0-19.7)	
Median iPFS, (month)	18.2 (95% CI: 12.5-23.8)	
Median PFS, (month)	8.9 (95% CI: 5.8-12.0)	
Median OS, (month)	17.8 (95% CI: 12.4-23.2)	
6-month OS (%)	89.1%	
6-month PFS (%)	67.5%	
1-year OS (%)	70.1%	
1-year PFS (%)	34.5%	
2‐year OS (%)	20.3%	

RECIST, Response Evaluation Criteria in Solid Tumors; mRECIST, modified RECIST; CR, complete response; PR, partial response; SD, stable disease; PD, progression disease; ORR, objective response rate; DCR, disease control rate; PFS, progression-free survival; iPFS, intrahepatic PFS; OS, overall survival; CI, confidence interval.

### Univariate and multivariate Cox regression analysis of prognostic factors

The prognostic factors were evaluated using Cox regression analysis, as detailed in **Supplementary Table 3**. Univariate Cox regression analysis revealed that higher Child-Pugh scores, lower levels of serum ALB and recurrent tumor were associated with increased risk of PFS, while responder based on RECIST 1.1 and mRECIST acted as protective factors for PFS. Univariate Cox regression analysis identified higher Child-Pugh scores, lower levels of serum ALB, levels of CRP>10 mg/L, the presence of lymph node metastasis and distant metastasis, late TNM stage and recurrent tumor as risk factors. Meanwhile, conversion to resection, responder based on RECIST 1.1 and mRECIST were protective factors for OS. Multivariate Cox regression analysis demonstrated that higher Child-Pugh scores (*p* = 0.011) and recurrent tumor (*p* = 0.021) were independent risk factors for PFS. Concurrently, multivariate Cox regression analysis for OS revealed that higher levels of CRP>10 mg/L (*p* = 0.014) and recurrent tumor (*p* = 0.028) were independent risk factors, while responder based on RECIST 1.1 (*p* = 0.006) was independent protective factor.

### Subgroup analysis

**Figure 3:**
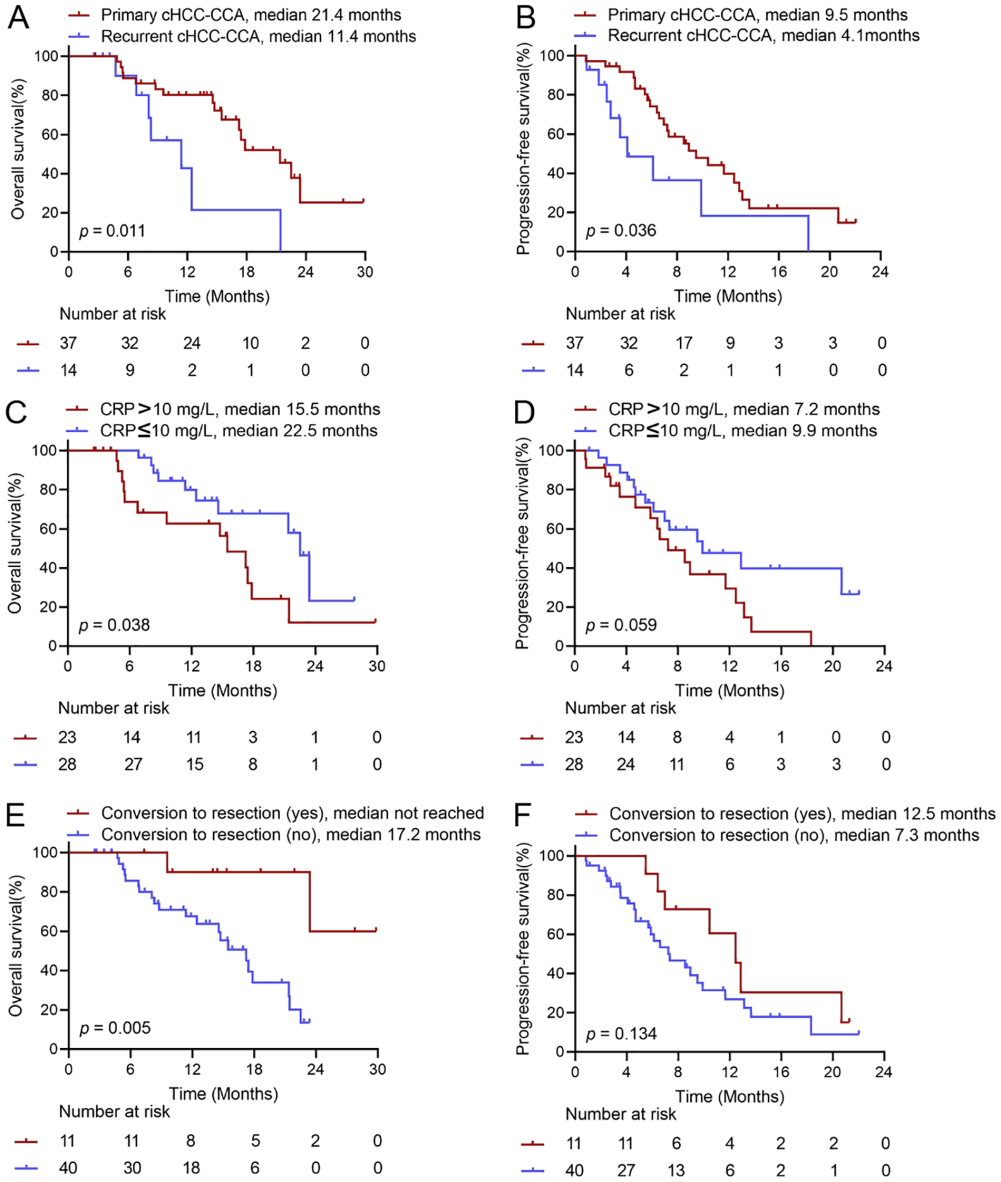
Subgroup analysis of overall survival and progression-free survival in patients treated with first-line interventional treatment combined with immunotargeted therapy. **(A, B)** Overall survival and progression-free survival were evaluated by Kaplan‐Meier curve, stratified by status of disease. **(C, D)** Overall survival and progression-free survival were evaluated by Kaplan‐Meier curve, stratified by C reactive protein level. **(E, F)** Overall survival and progression-free survival were evaluated by Kaplan‐Meier curve, stratified by whether accepting conversion to resection. cHCC-CCA, combined hepatocellular-cholangiocarcinoma; CRP, C-reactive protein.

Subgroups of ORR stratified by baseline characteristics were presented in **Supplementary Table 4**. When stratified by AFP levels, patients with AFP ≥ 400 ng/mL demonstrated a higher ORR than those with AFP < 400 ng/mL based on RECIST 1.1 (63.2% vs. 28.1%, *p* = 0.014) and mRECIST (78.9% vs. 43.8%, *p* = 0.014). Additionally, patients with primary cHCC-CCA exhibited a higher ORR compared to those with recurrent cHCC-CCA based on RECIST 1.1 (51.4% vs. 14.3%, *p* = 0.016). Furthermore, patients with macroscopic vein invasion have a significant higher ORR than those without macroscopic vein invasion according to RECIST 1.1 (58.3% vs. 25.9%, *p* = 0.019). Moreover, subgroups stratified by other baseline characteristics, including age, sex, HBsAg, ECOG, level of CA19-9, composition of the tumor, largest tumor size, tumor number, and TNM stage, presented similar ORRs. Patients with primary cHCC-CCA showed significantly prolonged OS (median OS: 21.4 months vs. 11.4 months, *p* = 0.011) and PFS (median PFS: 9.5 months vs. 4.1 months, *p* = 0.036) compared to those with recurrent cHCC-CCA (**Figures 3A, B**). The median OS for patients with CRP ≤ 10 mg/L was significantly longer than that for patients with CRP>10 mg/L (median OS: 22.5 months vs. 15.5 months, *p* = 0.038). A similar trend was observed in PFS, although it lacked statistical significance (median PFS: 9.9 months vs. 7.2 months, *p* = 0.059) (**Figures 3C, D**). The median OS for patients who underwent subsequent hepatic resection was significantly longer than for those who did not (median OS: not reached vs. 17.2 months, *p* = 0.005). A similar trend was noted in PFS, though it did not achieve statistical significance (median PFS: 12.5 months vs. 7.3 months, *p* = 0.134) (**Figures 3E, F**). When stratified by tumor composition and type of interventional treatment, the patients with dominant HCC did not show significant difference for OS (*p* = 0.835) and PFS (*p* = 0.553) compared to those with dominant iCCA (**Supplementary Figures 4A, B**), and the patients receiving TACE-HAIC treatment had similar OS (*p* = 0.713) and PFS (*p =* 0.868) compared to those receiving either TACE or HAIC treatment (**Supplementary Figures 4C, D**). Stratified by proportion of HCC component, ranging from ≥20%, 30%, 40%, 50%, 60%, 70%, 80%, no significant differences were observed in OS and PFS between groups (**Supplementary Figure 5**).

### Adverse events and safety

Adverse events were summarized in **Supplementary Table 5**. Overall, 50 patients (98.0%) experienced varying degrees of AEs. The most common AEs included elevated AST (54.9%), followed by hypoalbuminemia (49.0%), weight loss (39.2%), elevated ALT (35.3%), anemia (33.3%), decreased appetite (31.4%) and fatigue (31.4%), et al. Notably, 6 patients (11.8%) experienced grade ≥3 AEs, including leukopenia (n=1, 2.0%), neutropenia (n=1, 2.0%), thrombocytopenia (n=2, 3.9%), elevated ALT (n=2, 3.9%), elevated AST (n=2, 3.9%), hypoalbuminemia (n=2, 3.9%), hyperbilirubinemia (n=1, 2.0%). Immunotherapy was discontinued for 2 patients due to grade ≥3 elevations in ALT and AST. Immune-related pneumonitis occurred in 5 patients (9.8%), resulting in a dose delay of immunotherapy. There were no treatment-related deaths reported in this study.

## Discussion

This retrospective cohort study evaluated 51 patients with histologically confirmed unresectable cHCC-CCA receiving triple therapy (interventional treatment combined with immunotargeted therapy). The regimen demonstrated promising clinical efficacy, with a median OS of 17.8 months and a median PFS of 8.9 months. Notably, this study represents the first global report of this therapeutic approach for cHCC-CCA.

In recent years, several studies have demonstrated the efficacy and safety of interventional treatments (TACE or HAIC) combined with immunotargeted therapy for advanced HCC and advanced iCCA. He et al. conducted a multicenter study showing that advanced HCC patients who received HAIC plus lenvatinib and toripalimab achieved significantly better PFS and OS compared to those who received lenvatinib alone (27). The CHANCE001 trial reported that TACE combined with PD-(L)1 inhibitors and molecular targeted therapies significantly improved PFS, OS, and ORR in predominantly advanced HCC patients compared to TACE alone (12). Our previous study found that HAIC combined with lenvatinib and PD-(L)1 inhibitors resulted in significantly better OS, PFS and ORR compared to those receiving systemic chemotherapy for unresectable iCCA (14). Unfortunately, evidence from prospective phase III clinical trials regarding triple therapy for advanced HCC and advanced iCCA remains scared. Due to the rarity of cHCC-CCA, advancements in its treatment has been limited. Currently, evidence-based treatment options for cHCC-CCA have not been firmly established. Some retrospective studies have reported the efficacy of sorafenib or systemic chemotherapy for cHCC-CCA, with median OS ranged from 8.3 to 16.2 months and median PFS from 2.9 to 9.0 months (4, 5, 22, 28). These studies concluded that sorafenib and platinum-based chemotherapy exhibited a similar efficacy for cHCC-CCA. Although our study was a single-arm clinical study, it revealed that triple therapy achieved relatively better OS and PFS in cHCC-CCA patients compared to previous studies. The ICIs therapy has shown promising results in phase III clinical trials for HCC and iCCA patients. Nevertheless, only a few studies have assessed the effects of ICIs on cHCC-CCA. A small sample study, comprising 25 patients, who primarily received ICIs as second-line or later therapy, reported median PFS and OS of 3.5 months and 8.3 months, respectively (29). Rizell M documented a case of cHCC-CCA with lung metastases that achieved complete response following third-line treatment with pembrolizumab (30). Additionally, only one study has reported the efficacy of TACE for primary unresectable and recurrent cHCC-CCA, suggesting that efficacy was associated with tumor vascularity, with a median OS of 16 months for hypervascular tumors and 4 months for hypovascular tumors (31).

The favorable outcome of this triple therapy may be attributed to its synergistic antitumor effects. Firstly, HAIC enables for the delivery of higher concentrations of chemotherapeutic agents directly to the liver. These agents promote the maturation and function of dendritic cells and T cells, thereby activating the adaptive immune system (15). In the context of HCC, TACE can induce immunogenic cell death, transforming the immune microenvironment from immunosuppressive to immunogenic (16). TACE causes tumor tissue necrosis, reducing the release of immunosuppressive factors and mitigating immune function inhibition (17). Additionally, the necrotic tumor tissue can activate systemic immune responses by altering the phenotypes of peripheral immune cells (32). Secondly, the combination of ICIs with either TKIs or vascular endothelial growth factor (VEGF) inhibitors has shown potential synergistic effect in several studies. The underlying hypothesis suggests that TKIs induce the conversion of a nonimmunogenic ‘cold’ tumor into an inflamed ‘hot’ tumor by blocking MAPK, Wnt-β-catenin, CDK4-CDK6 or PTEN-dependent signaling pathways (33). Similarly, the inhibition of VEGF can alleviate immunosuppression, while immunotherapies can induce changes in the tumor exert anti-vascular effects. Thus, immunotherapy and/or anti-angiogenic therapies may create a cycle of immunostimulation and vascular remodeling within tumors (34). Thirdly, antiangiogenic therapy (e.g. antibodies targeting VEGF or TKIs) can delay the revascularization and recurrence of tumor after TACE (35). TKIs can promote tumor vascular normalization, which is expected to enhance response rates by improving the delivery of embolism agent and optimizing the embolization effect (36). In recent years, the use of sorafenib for HCC has declined due to the superior efficacy of lenvatinib (37), alongside the rising use of bevacizumab. In our study, the anti-angiogenic agents primarily included lenvatinib and bevacizumab.

Regarding safety, 50 out of 51 patients (98.0%) experienced at least one adverse event from any cause, with an occurrence rate of grade ≥3 AEs of 11.8%. The grade ≥3 AEs reported included leukopenia, neutropenia, thrombocytopenia, elevated ALT, elevated AST, hypoalbuminemia, and hyperbilirubinemia. Overall, the AEs observed in this study were predictable and manageable, primarily of mild-to-moderate severity. Only 2 patients discontinued ICI therapy due to grade ≥3 elevations in ALT and AST, while 5 patients delayed the dose of ICI therapy due to immune-related pneumonitis. In comparison, the IMbrave 150 and ORIENT-32 trials, which involved patients receiving atezolizumab plus bevacizumab or sintilimab plus a bevacizumab biosimilar, reported grade ≥3 AEs in 56.5% and 55.0% of patients, respectively (7, 8).

Additionally, the study identified a CRP level >10 mg/L as a significant factor associated with poor OS. Previous research has indicated that the CRAFITY score, which incorporates both CRP and AFP levels, serves as a superior prognostic predictor for HCC patients undergoing locoregional-immunotherapy (38). Patients with primary cHCC-CCA may derive greater benefits from this triple therapy compared to those with recurrent cHCC-CCA. These prognostic factors warrant further explored in clinical practice. Notably, 21.6% of the patients underwent subsequent hepatic resection due to tumor downstaging, and these individuals demonstrated improved survival. This triple therapy could potentially function as a conversion therapy for unresectable cHCC-CAA. A meta-analysis reported that the combination of lenvatinib with ICIs and locoregional therapy for unresectable HCC yielded a pooled conversion rates of 35% (95% CI: 23%-47%). However, it is essential to note that these results were derived from retrospective studies (39). In a prospective, single−arm and multicenter study involving 55 patients with unresectable HCC, 26 patients (47.3%) underwent surgery following successful conversion therapy with TACE combined with lenvatinib and camrelizumab. Nonetheless, long-term survival outcomes require extended follow-up for confirmation (40).

This study has several limitations. Firstly, this is a single-arm study without a control group, and 80.4% of the patients were hepatitis B virus positive, which may limit the generalizability of the results to the broader population. Secondly, this is a retrospective and single-center study, the findings should be verified through prospective and randomized controlled trials. Thirdly, the prognostic factors identified should be further validated in future study, due to the small sample size of our research. Fourthly, the varieties of PD-(L)1 inhibitors and anti-angiogenic drugs may affect the homogeneity of treatment procedures. Fifthly, interval for tumor response assessment might introduce variability. Finally, the assessment of drug toxicity may be underestimated because of the retrospective recording of adverse events outside of a clinical trial.

In conclusion, the triple combination therapy consisting of interventional treatment, PD-(L)1 inhibitor, and molecular targeted drug is an effective and safe option for treating unresectable cHCC-CCA. Our research provides data support for prospective and randomized controlled trials in the future.

## Data Availability

The original contributions presented in the study are included in the article/**Supplementary Material**. Further inquiries can be directed to the corresponding author.

## Supplementary material

Supplementary Figure 1 Representative images of cHCC-CCA. H&E and immunohistochemistry staining of cHCC-CCA by CK7 (CCA marker) and HepPar-1 (HCC marker). Magnification, 2×, scale bar = 1.25 mm. 10×, scale bar = 200 μm. 40×, scale bar = 50 μm. H&E, hematoxylin and eosin; CK7, cytokeratin 7; HepPar-1, hepatocyte paraffin 1; iCCA, intrahepatic cholangiocarcinoma; HCC, hepatocellular carcinoma.

Supplementary Figure 2 Waterfall plots for best change from baseline in intrahepatic target lesions. CR, complete response; PR, partial response; SD, stable disease; PD, progressive disease; RECIST, Response Evaluation Criteria In Solid Tumors; mRECIST, modified RECIST.

Supplementary Figure 3 Swimmer’s plot showing the clinical courses of 51 patients with unresectable combined hepatocellular-cholangiocarcinoma.

Supplementary Figure 4 Subgroup analysis of overall survival and progression-free survival in patients treated with first-line interventional treatment plus immunotargeted therapy. **(A, B)** Overall survival and progression-free survival were evaluated by Kaplan‐Meier curve, stratified by composition of the tumor; **(C, D)** Overall survival and progression-free survival were evaluated by Kaplan‐Meier curve, stratified by interventional treatment.

Supplementary Figure 5 Subgroup analysis of overall survival and progression-free survival in patients treated with first-line interventional treatment plus immunotargeted therapy. Overall survival and progression-free survival were evaluated by Kaplan‐Meier curve, stratified by HCC component with ≥20% **(A, B)**, ≥30% **(C, D)**, ≥40% **(E, F)**, ≥50% **(G, H)**, ≥60% **(I, J)**, ≥70% **(K, L)**, ≥80% (M-N). HCC, hepatocellular carcinoma.
