# Supplementary material for: Interventional treatment combined with immunotargeted therapy in unresectable combined hepatocellular-cholangiocarcinoma: a real-world retrospective cohort study
Source: Front Immunol. 2025 Jul 1;16:1591127. doi: 10.3389/fimmu.2025.1591127 (PMC12259696; doi:10.3389/fimmu.2025.1591127)
Supplement: Supplementary Figure 1 — Representative images of cHCC-CCA. H&E and immunohistochemistry staining of cHCC-CCA by CK7 (CCA marker) and HepPar-1 (HCC marker). Magnification, 2×, scale bar = 1.25 mm. 10×, scale bar = 200 μm. 40×, scale bar = 50 μm. H&E, hematoxylin and eosin; CK7, cytokeratin 7; HepPar-1, hepatocyte paraffin 1; iCCA, intrahepatic cholangiocarcinoma; HCC, hepatocellular carcinoma. [file Table1.docx]

Supplementary table 1. Summary of immunotargeted drugs for combined hepatocellular-cholangiocarcinoma.

| **Treatment** | **Drug name** | **Administration** | **Dose** | **Frequency** |
| --- | --- | --- | --- | --- |
| **Targeted therapy** | Lenvatinib | Oral | 8 mg, <60kg; 12mg, ≥60kg | QD |
|  | Apatinib | Oral | 750mg | QD |
|  | Sorafenib | Oral | 400mg | BID |
|  | Bevacizumab | Intravenous | 15mg/kg | Q3W |
| **Immunotherapy** | Toripalimab | Intravenous | 240 mg | Q3W |
|  | Sintilimab | Intravenous | 200 mg | Q3W |
|  | Tislelizumab | Intravenous | 200 mg | Q3W |
|  | Pembrolizumab | Intravenous | 200 mg | Q3W |
|  | Camrelizumab | Intravenous | 3mg/kg | Q3W |
|  | Atezolizumab | Intravenous | 1200mg | Q3W |
|  | Durvalumab | Intravenous | 1500 mg | Q3W |

**Abbreviations:** QD, once a day; BID, twice a day; Q3W, once every 3 weeks.

Supplementary table 2. Therapies in unresectable combined hepatocellular-cholangiocarcinoma

| **Therapies** | **N=51** |
| --- | --- |
| Interventional treatment |  |
| TACE, n (%) | 10 (19.6) |
| HAIC, n (%) | 26 (51.0) |
| TACE-HAIC, n (%) | 15 (29.4) |
| Interventional treatment cycles | 3 (2-5) |
| Targeted therapy |  |
| Lenvatinib, n (%) | 38 (74.5) |
| Apatinib, n (%) | 4 (7.8) |
| Sorafenib, n (%) | 1 (2.0) |
| Bevacizumab, n (%) | 8 (15.7) |
| Duration of targeted therapy, months | 5.8 (3.0-11.7) |
| Immunotherapy |  |
| Toripalimab | 3 (5.9) |
| Sintilimab | 17 (33.3) |
| Tislelizumab | 10 (19.6) |
| Pembrolizumab | 7 (13.7) |
| Camrelizumab | 8 (15.7) |
| Atezolizumab | 5 (9.8) |
| Durvalumab | 1 (2.0) |
| Immunotherapy cycles | 5 (3-9) |

**Abbreviations:** cHCC-CCA, combined hepatocellular-cholangiocarcinoma; TACE, transarterial chemoembolization; HAIC, hepatic arterial infusion chemotherapy, TACE-HAIC, TACE combined with HAIC.

Supplementary table 3. Cox regression analysis of prognostic factors for progression-free survival and overall survival

| **Variables** | **Progression-free survival** | | | | **Overall survival** | | | |
| --- | --- | --- | --- | --- | --- | --- | --- | --- |
|  | **Univariate** | | **Multivariate** | | **Univariate** | | **Multivariate** | |
|  | **HR (95% CI)** | ***p value*** | **HR (95% CI)** | ***p value*** | **HR (95%CI)** | ***p value*** | **HR (95% CI)** | ***p value*** |
| Age (≥55/<55) | 0.58(0.29-1.16) | 0.122 |  |  | 0.95 (0.42-2.16) | 0.898 |  |  |
| Gender (Male/Female) | 21.53(0.01->1000) | 0.486 |  |  | 0.38 (0.05-2.89) | 0.346 |  |  |
| HbsAg (Positive/Negative | 2.23(0.78-6.35) | 0.135 |  |  | 0.93 (0.34-2.54) | 0.889 |  |  |
| ECOG (1/0) | 1.40(0.70-2.78) | 0.340 |  |  | 1.23(0.54-2.81) | 0.627 |  |  |
| Child-Pugh (6/5) | 3.15(1.49-6.67) | 0.003 | 3.70 (1.35-10.14) | 0.011 | 3.56(1.38-9.22) | 0.009 | 2.87(0.74-11.1) | 0.126 |
| ALB (<40/≥40 g/L) | 2.38(1.16-4.86) | 0.018 | 1.38 (0.53-3.57) | 0.507 | 3.25 (1.35-7.83) | 0.009 | 0.26(0.04-1.79) | 0.172 |
| TBIL (>17.1/≤17.1μmol/L) | 1.02(0.49-2.12) | 0.953 |  |  | 1.29(0.55-3.00) | 0.558 |  |  |
| CRP (>10/≤10 mg/L) | 1.71(0.85-3.43) | 0.133 |  |  | 2.37(1.03-5.46) | 0.044 | 19.9(1.82-217.42) | 0.014 |
| AFP (≥400/<400 ng/mL) | 0.77(0.38-1.57) | 0.472 |  |  | 0.63(0.26-1.53) | 0.310 |  |  |
| PIVKA-II (>40/≤40mAU/mL) | 1.60(0.66-3.88) | 0.301 |  |  | 1.07(0.42-2.71) | 0.893 |  |  |
| CA 19-9 (>35/≤35U/mL) | 1.40(0.69-2.83) | 0.347 |  |  | 1.15(0.51-2.63) | 0.735 |  |  |
| Liver cirrhosis (Present/Absent) | 0.91(0.45-1.85) | 0.802 |  |  | 1.07(0.46-2.53) | 0.870 |  |  |
| Composition of tumor (HCC dominant/iCCA dominant) | 1.26(0.58-2.73) | 0.554 |  |  | 1.09(0.47-2.54) | 0.835 |  |  |
| Largest tumor size (>5/≤5cm) | 1.54(0.66-3.62) | 0.317 |  |  | 1.02(0.38-2.77) | 0.968 |  |  |
| Tumor numbers (Multiple/Solitary) | 1.73(0.67-4.49) | 0.262 |  |  | 0.84(0.28-2.50) | 0.749 |  |  |
| Macroscopic vein invasion (Present/Absent) | 0.82(0.41-1.63) | 0.567 |  |  | 0.86(0.37-1.98) | 0.714 |  |  |
| Lymph node metastasis (Present/Absent) | 1.58(0.79-3.15) | 0.195 |  |  | 2.88(1.18-7.02) | 0.020 | 2.82(0.43-18.32) | 0.278 |
| Distant metastasis (Present/Absent) | 1.60(0.74-3.49) | 0.236 |  |  | 3.12(1.33-7.35) | 0.009 | 2.33(0.53-10.36) | 0.265 |
| TNM stage (IV/II-III) | 1.23(0.61-2.50) | 0.563 |  |  | 5.03(1.48-17.07) | 0.010 | 0.55(0.04-7.84) | 0.659 |
| Status of disease (Recurrent/Primary) | 2.26(1.03-4.96) | 0.041 | 2.95(1.17-7.39) | 0.021 | 3.19(1.25-8.15) | 0.015 | 5.03(1.19-21.21) | 0.028 |
| Conversion to resection (Yes/No) | 0.53(0.23-1.23) | 0.141 |  |  | 0.10(0.01-0.73) | 0.024 | 0.18(0.01-2.33) | 0.187 |
| Interventional therapy (TACE-HAIC/TACE or HAIC) | 0.81(0.38-1.74) | 0.589 |  |  | 1.20(0.46-3.13) | 0.713 |  |  |
| Interventional therapy cycle (≥3/<3) | 1.63(0.80-3.35) | 0.182 |  |  | 0.82(0.35-1.91) | 0.650 |  |  |
| Immunotherapy cycle (≥5/<5) | 0.72(0.36-1.46) | 0.365 |  |  | 0.72(0.31-1.68) | 0.446 |  |  |
| Duration of targeted therapy (≥6/<6 months) | 0.56(0.28-1.12) | 0.101 |  |  | 0.49(0.21-1.13) | 0.096 |  |  |
| Response (RECIST 1.1, responder/non-responder) | 0.32(0.15-0.68) | 0.003 | 0.55(0.16-1.85) | 0.332 | 0.26(0.10-0.67) | 0.005 | 0.04(0.01-0.41) | 0.006 |
| Response (mRECIST, responder/non-responder) | 0.29(0.14-0.60) | 0.001 | 0.39(0.13-1.22) | 0.107 | 0.30(0.13-0.70) | 0.005 | 1.14(0.23-5.61) | 0.876 |

**Abbreviations:** HR, hazard ratio; CI: confidence interval; HBsAg, hepatitis B surface antigen; ECOG, Eastern Cooperative Oncology Group; ALB, albumin; TBIL, total bilirubin; CRP, C-reactive protein; AFP, alpha-fetoprotein; PIVKA-II, protein induced by vitamin K absence or antagonist-II; CA19–9, carbohydrate antigen 19-9; HCC, hepatocellular carcinoma; iCCA, intrahepatic cholangiocarcinoma; TNM, tumor–node–metastasis; TACE, transarterial chemoembolization; HAIC, hepatic arterial infusion chemotherapy; TACE-HAIC, TACE combined with HAIC; ;RECIST, Response Evaluation Criteria in Solid Tumors; mRECIST, modified RECIST

Supplementary table 4. ORR of patients in the subgroup according to baseline characteristics

| **Subgroup** | **RECIST 1.1** | | |  | **mRECIST** | | |
| --- | --- | --- | --- | --- | --- | --- | --- |
|  | **No. of PR or CR/total no. of patients** | **ORR, %** | ***p* value** |  | **No. of PR or CR/total no. of patients** | **ORR, %** | ***p* value** |
| All | 21/51 | 41.2 | - |  | 29/51 | 56.9 | - |
| Age |  |  | 0.688 |  |  |  | 0.886 |
| <55 years | 11/25 | 44.0 |  |  | 13/25 | 52.0 |  |
| ≥55 years | 10/26 | 38.5 |  |  | 13/26 | 50.0 |  |
| Sex |  |  | 0.506 |  |  |  | 0.181 |
| Male | 21/49 | 42.9 |  |  | 29/49 | 59.2 |  |
| Female | 0/2 | 0.0 |  |  | 0/2 | 0.0 |  |
| HBsAg |  |  | 0.658 |  |  |  | 0.398 |
| Positive | 18/41 | 43.9 |  |  | 25/41 | 61.0 |  |
| Negative | 3/10 | 30.0 |  |  | 4/10 | 40.0 |  |
| ECOG |  |  | 0.708 |  |  |  | 0.265 |
| 0 | 13/30 | 43.3 |  |  | 19/30 | 63.3 |  |
| 1 | 8/21 | 38.2 |  |  | 10/21 | 47.6 |  |
| Chlid-Pugh |  |  | 0.377 |  |  |  | 0.366 |
| 5 | 17/38 | 44.7 |  |  | 23/38 | 60.5 |  |
| 6 | 4/13 | 30.8 |  |  | 6/13 | 46.2 |  |
| ALB (g/L) |  |  | 0.546 |  |  |  | 0.110 |
| < 40 | 6/17 | 35.3 |  |  | 7/17 | 41.2 |  |
| ≥ 40 | 15/34 | 44.1 |  |  | 22/34 | 64.7 |  |
| TBIL (μmol/L) |  |  | 0.139 |  |  |  | 0.246 |
| > 17.1 | 9/16 | 56.3 |  |  | 11/16 | 68.8. |  |
| ≤17.1 | 12/35 | 34.2 |  |  | 18/35 | 51.4 |  |
| CRP (mg/L) |  |  | 0.762 |  |  |  | 0.540 |
| > 10 | 10/23 | 43.5 |  |  | 12/23 | 52.2 |  |
| ≤ 10 | 11/28 | 39.3 |  |  | 17/28 | 60.7 |  |
| AFP (ng/ml) |  |  | 0.014 |  |  |  | 0.014 |
| ≥ 400 | 12/19 | 63.2 |  |  | 15/19 | 78.9 |  |
| < 400 | 9/32 | 28.1 |  |  | 14/32 | 43.8 |  |
| PIVKA-II (mAU/ml) |  |  | 0.174 |  |  |  | 0.117 |
| > 40 | 17/36 | 47.2 |  |  | 23/36 | 63.9 |  |
| ≤ 40 | 4/15 | 26.7 |  |  | 6/15 | 40.0 |  |
| CA19-9 (U/ml) |  |  | 0.762 |  |  |  | 0.964 |
| > 35 | 10/23 | 43.5 |  |  | 13/23 | 56.5 |  |
| ≤ 35 | 11/28 | 39.3 |  |  | 16/28 | 57.1 |  |
| Liver cirrhosis |  |  | 0.059 |  |  |  | 0.169 |
| Present | 16/31 | 51.6 |  |  | 20/31 | 64.5 |  |
| Absent | 5/20 | 25.0 |  |  | 9/20 | 45.0 |  |
| Composition of the tumor^#^ |  |  | 0.169 |  |  |  | 0.135 |
| HCC dominant | 15/29 | 51.7 |  |  | 20/29 | 69.0 |  |
| iCCA dominant | 6/19 | 31.6 |  |  | 9/19 | 47.4 |  |
| Largest tumor size (cm) |  |  | 0.261 |  |  |  | 0.543 |
| > 5 | 17/37 | 45.9 |  |  | 22/37 | 59.5 |  |
| ≤ 5 | 4/14 | 28.6 |  |  | 7/14 | 50.0 |  |
| Tumor number |  |  | 0.368 |  |  |  | 0.930 |
| Solitary | 2/9 | 22.2 |  |  | 5/9 | 55.6 |  |
| Multiple | 19/42 | 45.2 |  |  | 24/42 | 57.1 |  |
| Macroscopic vein invasion |  |  | 0.019 |  |  |  | 0.183 |
| Present | 14/24 | 58.3 |  |  | 16/24 | 66.7 |  |
| Absent | 7/27 | 25.9 |  |  | 13/27 | 48.1 |  |
| Lymph node metastasis |  |  | 0.332 |  |  |  | 0.313 |
| Present | 9/26 | 34.6 |  |  | 13/26 | 50.0 |  |
| Absent | 12/25 | 48.0 |  |  | 16/25 | 64.0 |  |
| Distant metastasis |  |  | 0.125 |  |  |  | 0.121 |
| Present | 3/13 | 23.1 |  |  | 5/13 | 38.5 |  |
| Absent | 18/38 | 47.4 |  |  | 24/38 | 63.2 |  |
| TNM stage |  |  | 0.200 |  |  |  | 0.199 |
| II-III | 10/19 | 52.6 |  |  | 13/19 | 68.4 |  |
| IV | 11/32 | 34.4 |  |  | 16/32 | 50.0 |  |
| Status of disease |  |  | 0.016 |  |  |  | 0.061 |
| Primary cHCC-CCA | 19/37 | 51.4 |  |  | 24/37 | 64.9 |  |
| Recurrent cHCC-CCA | 2/14 | 14.3 |  |  | 5/14 | 35.7 |  |
| Interventional therapy |  |  | 0.912 |  |  |  | 0.125 |
| TACE-HAIC | 6/15 | 40.0 |  |  | 11/15 | 73.3 |  |
| TACE or HAIC | 15/36 | 41.7 |  |  | 18/36 | 50.0 |  |

**Note:** ^#^Three patients were classified as neither HCC dominant nor iCCA dominant.

**Abbreviations:** ORR, objective response rate; RECIST, Response Evaluation Criteria in Solid Tumors; mRECIST, modified RECIST; CR, complete response; PR, partial response; HBsAg, hepatitis B surface antigen; ECOG, Eastern Cooperative Oncology Group; ALB, albumin; TBIL, total bilirubin; CRP, C-reactive protein; AFP, alpha-fetoprotein; PIVKA-II, protein induced by vitamin K absence or antagonist-II; CA19-9, carbohydrate antigen 19-9; HCC, hepatocellular carcinoma; iCCA, intrahepatic cholangiocarcinoma; TNM, tumor–node–metastasis; TACE, transarterial chemoembolization; HAIC, hepatic arterial infusion chemotherapy; TACE-HAIC, TACE combined with HAIC.

Supplementary table 5. Adverse events.

| **Adverse events** | **All patients (n = 51)** | | | |
| --- | --- | --- | --- | --- |
|  | **Any grade** |  | **Grade****≥ 3** | |
| Total | 50 (98.0) |  | 6 (11.8) | |
| Treatment-related AEs, n (%) |  |  |  | |
| Rash | 2 (3.9) |  | 0 (0.0) |  |
| Fever | 8 (15.7) |  | 0 (0.0) |  |
| Abdominal pain | 15 (29.4) |  | 0 (0.0) |  |
| Diarrhea | 7 (13.7) |  | 0 (0.0) |  |
| Vomiting | 2 (3.9) |  | 0 (0.0) |  |
| Decreased appetite | 16 (31.4) |  | 0 (0.0) |  |
| Fatigue | 16 (31.4) |  | 0 (0.0) |  |
| Sensory neuropathy | 0 (0.0) |  | 0 (0.0) |  |
| Weight loss | 20 (39.2) |  | 0 (0.0) |  |
| Hypertension | 13 (25.4) |  | 0 (0.0) |  |
| Laboratory-related AEs, n (%) |  |  |  |  |
| Leukopenia | 5 (9.8) |  | 1 (2.0) |  |
| Neutropenia | 4 (7.8) |  | 1 (2.0) |  |
| Anemia | 17 (33.3) |  | 0 (0.0) |  |
| Thrombocytopenia | 7 (13.7) |  | 2 (3.9) |  |
| Hyponatremia | 12 (23.5) |  | 0 (0.0) |  |
| Hypokalemia | 3 (5.9) |  | 0 (0.0) |  |
| Elevated ALT | 18 (35.3) |  | 2 (3.9) |  |
| Elevated AST | 28 (54.9) |  | 2 (3.9) |  |
| Hypoalbuminemia | 25 (49.0) |  | 2 (3.9) |  |
| Hyperbilirubinemia | 9 (17.6) |  | 1 (2.0) |  |
| Elevated creatinine | 4 (7.8) |  | 0 (0.0) |  |
| Immune-related AEs, n (%) |  |  |  |  |
| Allergic reaction | 2 (3.9) |  | 0 (0.0) |  |
| Dyspnea | 1 (2.0) |  | 0 (0.0) |  |
| Mucositis | 0 (0.0) |  | 0 (0.0) |  |
| Hyperthyroidism | 1 (2.0) |  | 0 (0.0) |  |
| Pneumonitis | 5 (9.8) |  | 0 (0.0) |  |
| Arthritis | 0 (0.0) |  | 0 (0.0) |  |
| Nephritis | 2 (3.9) |  | 0 (0.0) |  |
| Pruritus | 0 (0.0) |  | 0 (0.0) |  |
| Myocarditis | 0 (0.0) |  | 0 (0.0) |  |
| Enteritis | 0 (0.0) |  | 0 (0.0) |  |
| Uveitis | 0 (0.0) |  | 0 (0.0) |  |

**Abbreviations:** AEs, adverse events; ALT, alanine transaminase; AST, aspartate transaminase.


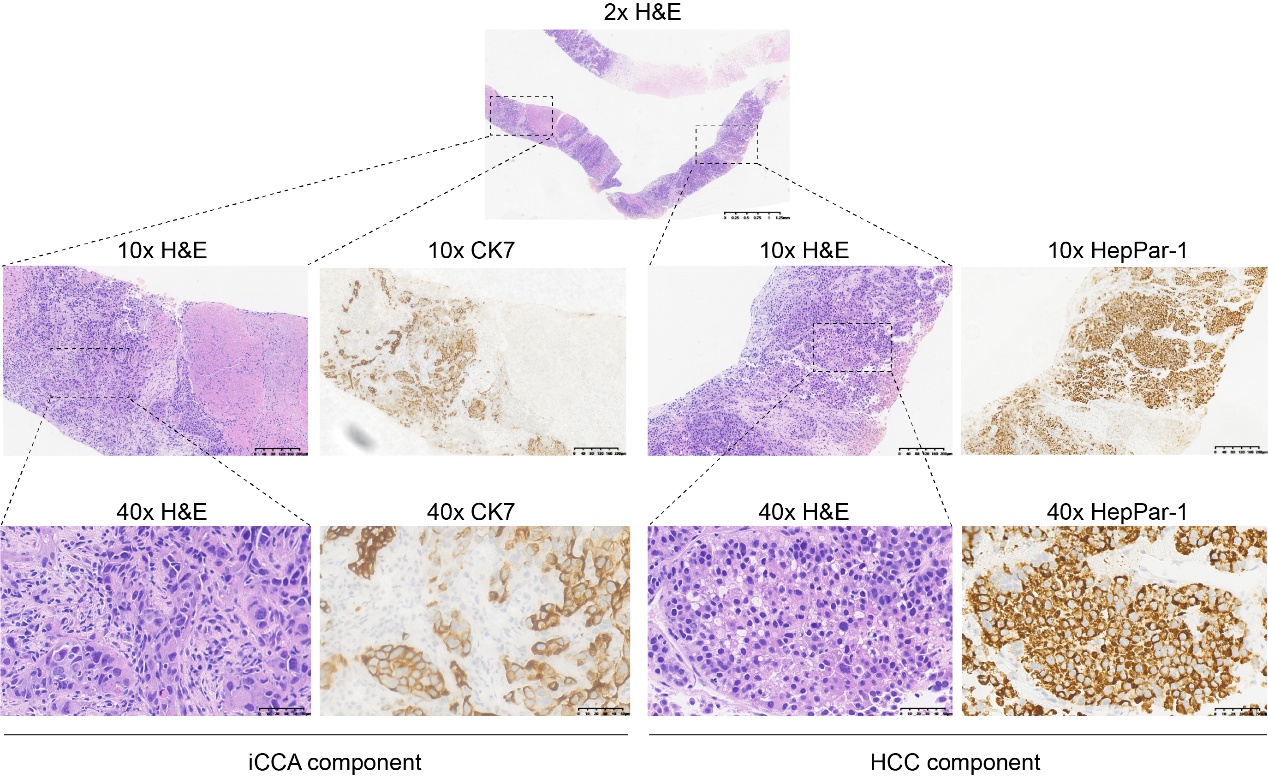


**Supplementary figure 1.** Representative images of cHCC-CCA. H&E and immunohistochemistry staining of cHCC-CCA by CK7 (CCA marker) and HepPar-1 (HCC marker). Magnification, 2×, scale bar = 1.25 mm. 10×, scale bar = 200 μm. 40×, scale bar = 50 μm. H&E, hematoxylin and eosin; CK7, cytokeratin 7; HepPar-1, hepatocyte paraffin 1; iCCA, intrahepatic cholangiocarcinoma; HCC, hepatocellular carcinoma.


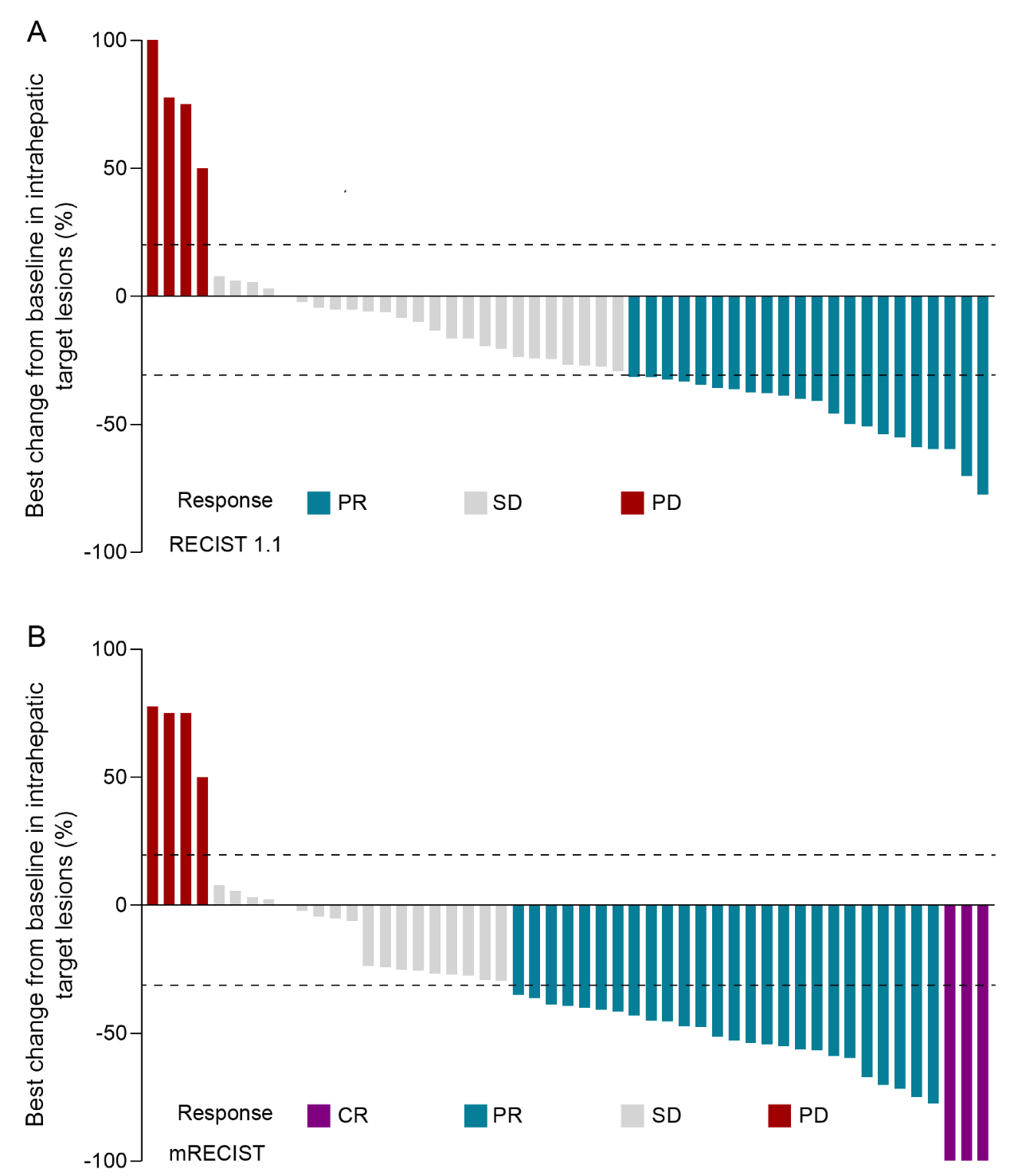


**Supplementary figure 2**. Waterfall plots for best change from baseline in intrahepatic target lesions. CR, complete response; PR, partial response; SD, stable disease; PD, progressive disease, RECIST, Response Evaluation Criteria In Solid Tumors; mRECIST, modified RECIST.


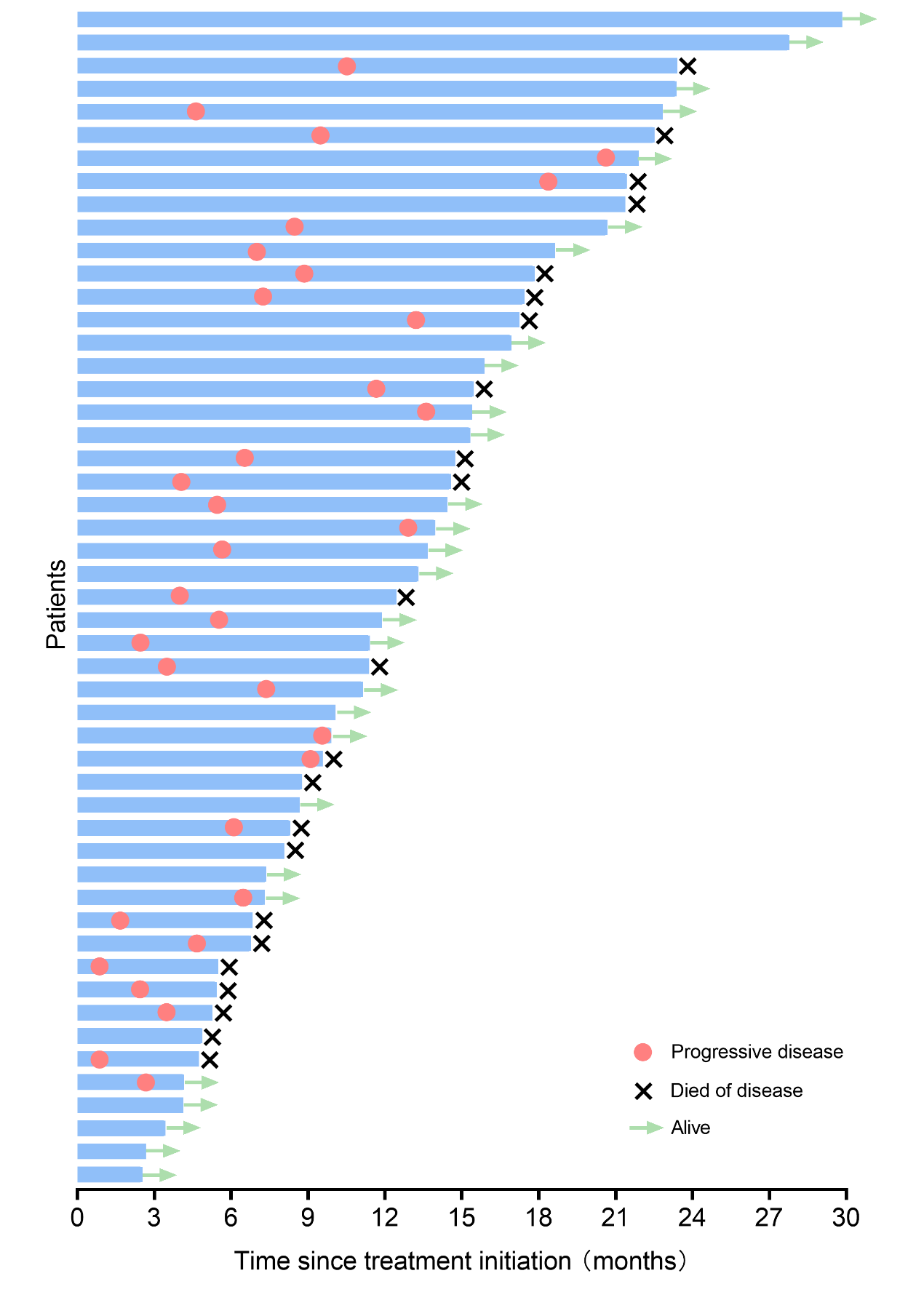


**Supplementary figure 3**. Swimmer’s plot showing the clinical courses of 51 patients with unresectable combined hepatocellular-cholangiocarcinoma.


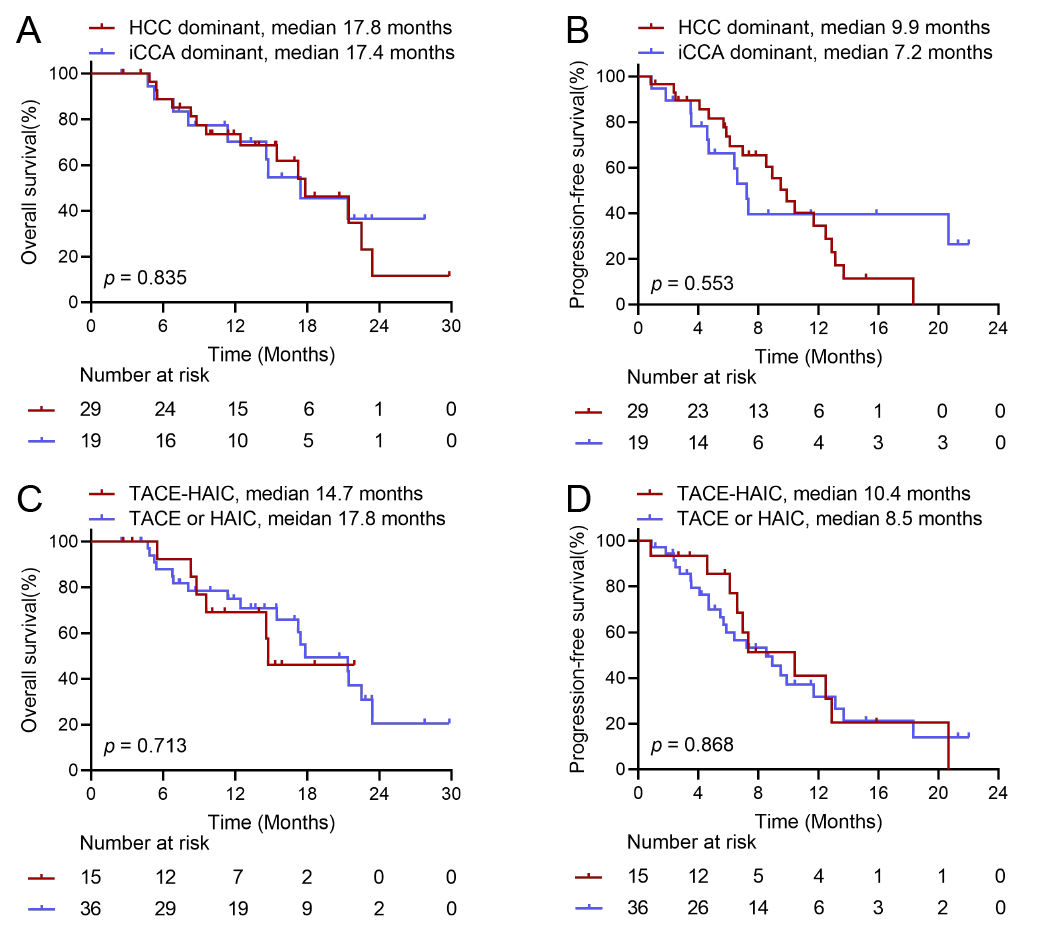


**Supplementary figure 4.** Subgroup analysis of overall survival and progression-free survival in patients treated with first-line interventional treatment plus immunotargeted therapy. (A-B) Overall survival and progression-free survival were evaluated by Kaplan‐Meier curve, stratified by composition of the tumor; (C-D) Overall survival and progression-free survival were evaluated by Kaplan‐Meier curve, stratified by interventional treatment.


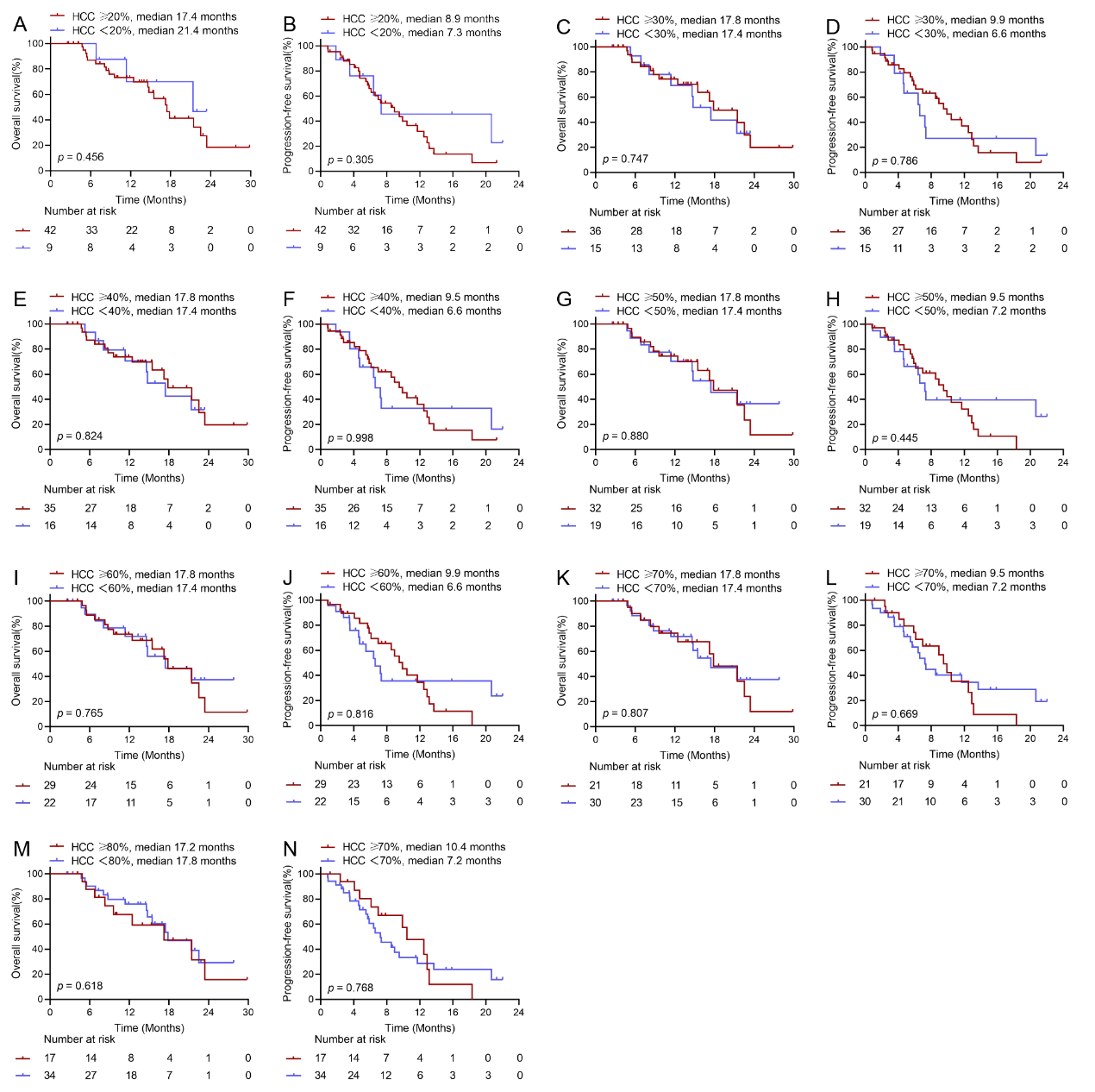


**Supplementary figure 5. s**ubgroup analysis of overall survival and progression-free survival in patients treated with first-line interventional treatment plus immunotargeted therapy. Overall survival and progression-free survival were evaluated by Kaplan‐Meier curve, stratified by HCC component with ≥20% (A-B), ≥30% (C-D), ≥40% (E-F), ≥50% (G-H), ≥60% (I-J), ≥70% (K-L), ≥80% (M-N). HCC, hepatocellular carcinoma.


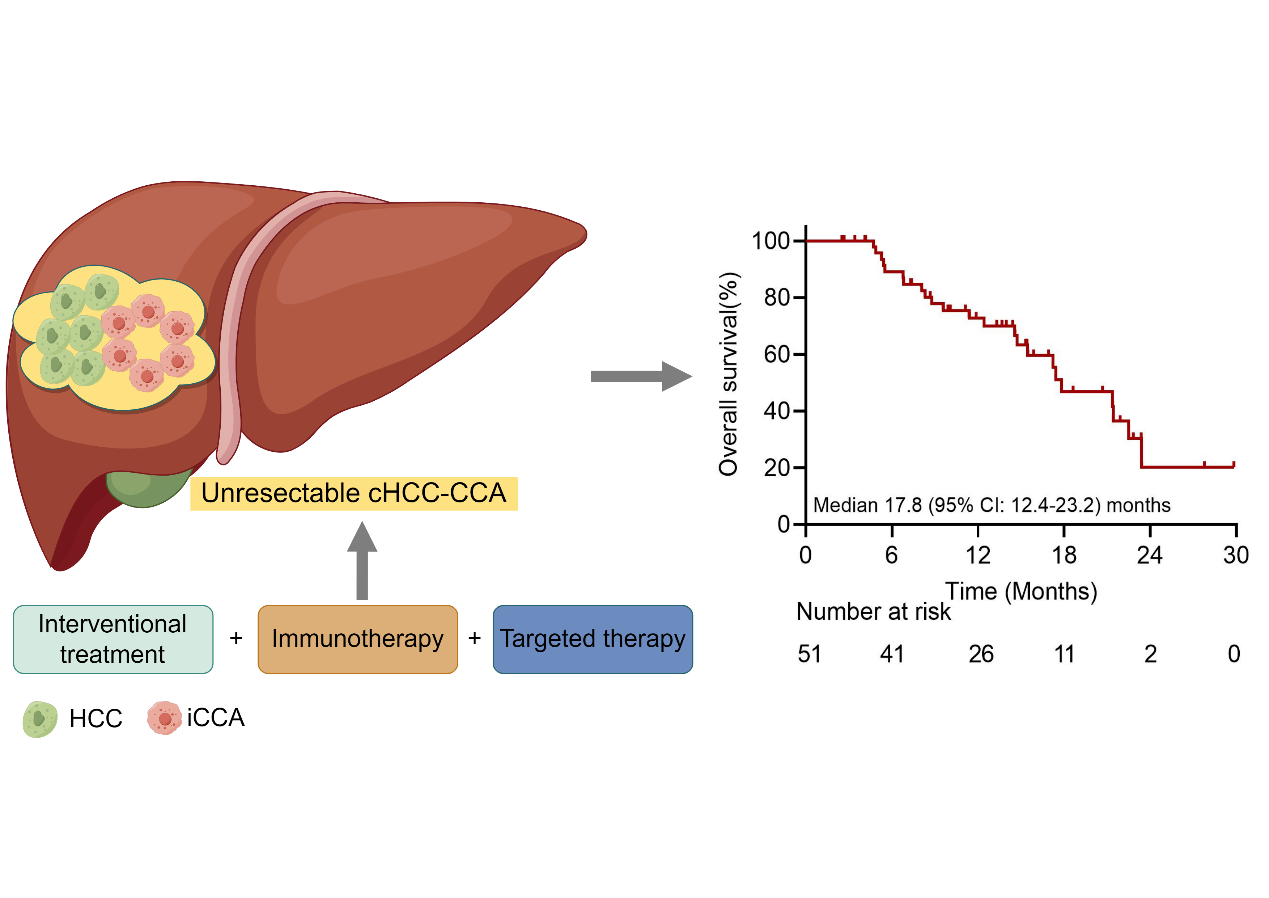


**Supplementary figure 6.** Graphical abstract
